# Exploring mothers’ experiences of accessing an Education Health and Care Plan (EHCP) for an autistic child attending mainstream school in the United Kingdom

**DOI:** 10.1080/20473869.2023.2298606

**Published:** 2024-02-09

**Authors:** Saskia Keville, Morgan Mills, Amanda K. Ludlow

**Affiliations:** Department of Psychology, Sport and Geography, University of Hertfordshire, Hatfield, UK

**Keywords:** Autism, Education, Health and Care Plan (EHCP), inclusivity, mainstream education, children, mothers, support, wellbeing

## Abstract

Many autistic children are educated in mainstream settings, yet despite the benefits it can present challenges for the children and their parents. A legal framework for accessing support for school age children in England are Education, Health and Care Plans (EHCP). Whilst there are anecdotal accounts on accessing EHCPs for autistic children, research is limited. To expand knowledge, six mothers (aged between 31 and 44) of autistic children (aged between 6 and 15) were interviewed about their EHCP experiences. A reflexive thematic analysis resulted in the following themes: Barriers for accessing educational support; the process of obtaining an EHCP; impact on mothers; trying to find glimmers of hope. The main finding was an intimidating and overwhelmingly difficult process mothers had to navigate to access an EHCP. The ensuing battle left them with feelings of isolation, anxiety, and hopelessness; alongside a sense that the systems set up to help were found to work against them. Nevertheless, some mothers were determined to find glimmers of hope for the benefit of their child’s development. For autistic children to access the benefits of mainstream education, wider systemic changes are urgently needed. This would also vicariously support parental wellbeing.

## Introduction

Around 1.8% of children in English schools are recognized as being autistic (Roman-Urrestarazu *et al.* 2021), with a large proportion of children attending mainstream education. Whilst many autistic children can have a positive education experience in mainstream settings, some of their needs are reported as being unmet (Humphrey and Lewis 2008). For example, autistic children will often show a very diverse profile in their academic achievement, performing both below and above expected levels. Yet many schools fail to respond to individual strengths, peaks and/or dips in academic learning (Keen *et al.* 2016).

Despite the drive for inclusivity in education, research consistently shows parents believe there is insufficient support in place for autistic children in mainstream settings (Galpin *et al.* 2018); with 74% of parents and carers believing schools in the United Kingdom do not meet their child’s needs (National Autistic Society (NAS) 2021). This is particularly troubling given in 2020, over 70% of the 82,847 autistic pupils in England were reported as being educated in mainstream provisions (Department for Education (DfE) 2020). Compounding this, many parents report dissatisfaction with teachers’ understanding of autism and, when surveyed, almost half of teachers reported a lack confidence in providing the necessary support for autistic children (NAS 2017). Thus, teachers may not always have appropriate autism awareness and/or knowledge to facilitate inclusive education (Martin *et al.* 2021).

Autism forms the largest group to be classed as a Special Educational Need (SEN), and support for children with SEN is crucial in managing educational and classroom demands. However, data from the DfE (2021) highlights potential disparities in outcomes for children with SEN support compared to those without SEN, potentially related to greater needs/disability. For example, young people with SEN are significantly less likely to progress to higher education than those without SEN (DfE 2021). Nevertheless, children with SEN, appear to struggle more with attendance demands in education. For example, in England it was found that children with SEN support are more likely to be persistently absent from school (17.9% with SEN support compared to 9% for pupils without SEN; perhaps indicative of more complex health needs); and significantly more likely to be permanently excluded from school (0.32% with SEN support compared to 0.06% without SEN). Indeed, children with SEN account for just under half of all permanent exclusions and suspensions within England’s state education system (GOV.UK 2022).

**Figure 1.**
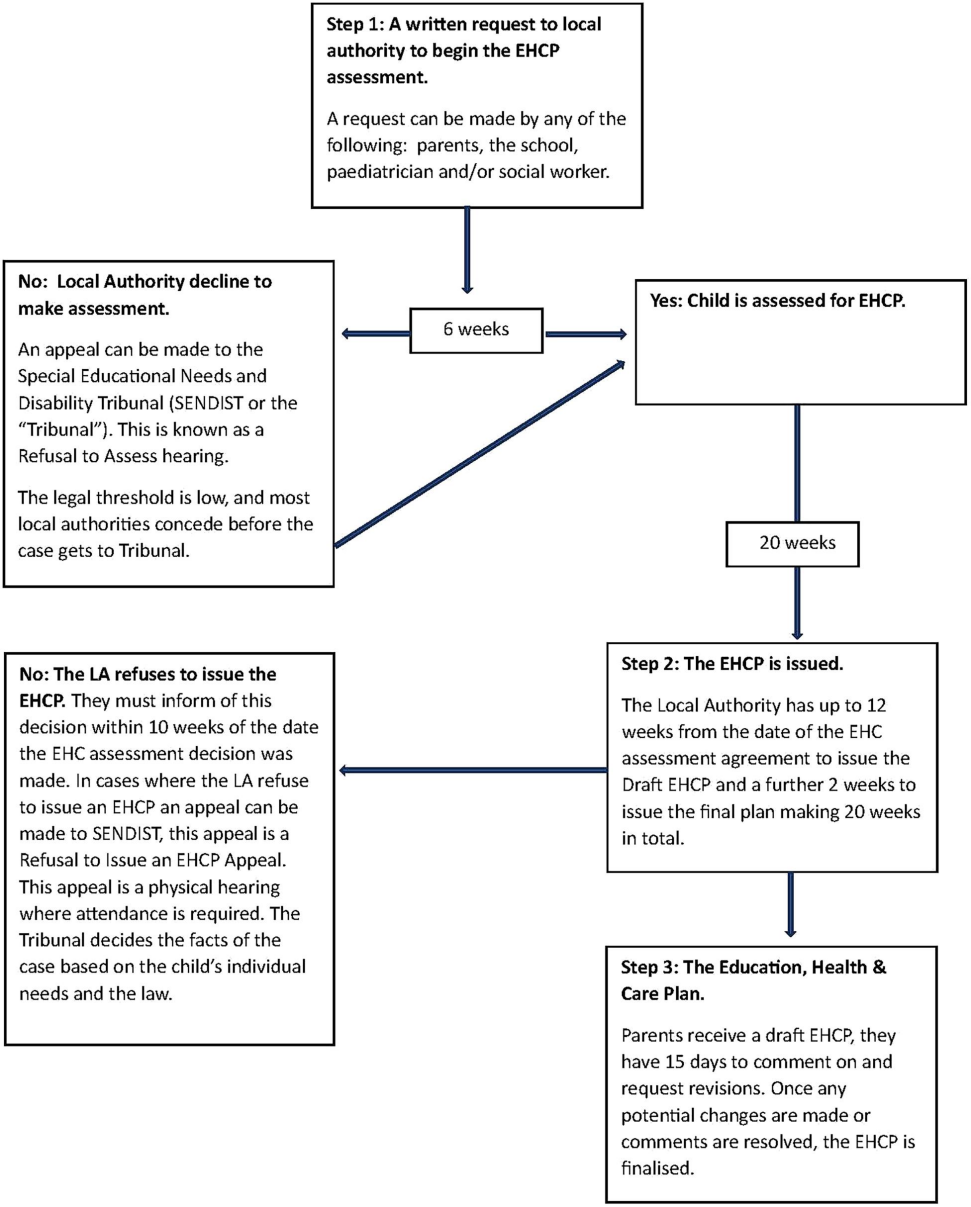
Outline of the EHCP process. EHCP: Educational Health and Care Plan.

When a child or young person up to age 25 has more learning needs which require additional educational support, parents can apply to the regional Local Authority (LA) for an assessment to access an Education, Health and Care Plan (EHCP) to legally meet their educational, health and social needs (GOV.UK 2023). An EHCP gives a child the individual support they need to meet their SEN, going beyond what the school can offer and providing additional resources to improve the quality of their learning experience. The EHCP states the provision that a child needs and must be provided irrespective of any local authorities funding issues. Importantly, the EHCP is a legally binding document and is enforceable through Judicial Review. The EHCP runs up until a child finishes education or turns 25 (for example, college, internship), whichever comes first, although it does not cover university education. An EHCP annual review takes place at least once a year and based on this review, which involves the parents, the school/college, and the local authority, allows complaints and revisions to be considered. The LA may seek to Cease to Maintain (stop) the EHCP, when the progress has been such that the child no longer needs the support of the EHCP (Wolfe and Glenister 2020). The process and duration of obtaining an EHCP is outlined in Figure 1.

Whilst there is a clear pathway with timelines to obtaining the EHCP parents can encounter setbacks with 42% being refused an EHCP assessment on their first request (NAS 2017). A recent report concluded that only 25% of parents were satisfied with the EHCP support their child received, with half of parents dissatisfied with the process of EHCPs, and 26% of parents waiting over three years for support (NAS 2021). There has been an annual increase in those gaining an EHCP since replacing statements in 2010 (DfE 2021); further, during 2022 there were 114,457 initial requests made for an EHC assessment, up from 93,300 in 2021–the highest number since data was first collected in 2016 (DfE 2023). Autism is amongst the most prevalent SEN group with an EHCP or statement (of the 294,800 pupils with EHCPs, 30% are autistic; DfE 2020); it is also identified as the most common need in tribunal appeals, accounting for 50% of all cases (DfE 2023).

While positive experiences of EHCPs have been noted with families feeling part of the decision-making process (Craston *et al.* 2015), and that their child’s EHCP plan led to accessing the necessary support (DfE 2017), more recent accounts have been less favorable (NAS 2021, Starkie 2023). Moreover, families who have either failed to secure an EHCP and/or are currently undergoing assessment have largely been neglected from the literature (Boesley and Crane 2018); potentially meaning some of the more negative experiences have not yet been captured.

Furthermore, school SEN coordinators (SENCOs), who play an increasingly pivotal role in the implementation of EHCPs (Curran *et al.* 2017), have highlighted the difficulties families have in obtaining an EHCP for their child (Palikara *et al.* 2019). For example, it was felt that only the children perceived as having more complex needs would be able to secure an EHCP, whereas for those children meeting age-related expectations in mainstream school this would be near impossible (Boesley and Crane 2018).

There has been limited research addressing parents’ experience of obtaining and managing EHCPs, especially those who have been unsuccessful or who are still undertaking the process. Given parents of autistic children already experience higher stress compared to parents who do not have an autistic child (Hayes and Watson 2013), struggling to access support can exacerbate their stressors and wellbeing (Brown *et al.* 2012). This may be particularly pertinent when accessing educational support for an autistic child, given teachers and schools must then manage these provisions. With support lowering stress and enhancing quality of life for parents (Elder *et al.* 2017), more effectively addressing the support needs of their child in school could vicariously support parents’ own wellbeing. Therefore, parents’ lived experiences of obtaining an EHCP for an autistic child attending mainstream school was addressed in the current study. Insight into the impact of the EHCP process for families of autistic children, based on their personal accounts and reflections, could help to identify ways to deliver better services to those in need.

## Methods

A qualitative exploratory design using thematic analysis (TA) was chosen to enable the researchers to delve deeper into experiences, explore similarities and differences within data, and use a reflexive approach to refine the codes and themes allowing a thorough exploration and a deeper understanding of the data (Braun and Clarke 2006, 2019).

### Procedure

Purposeful sampling was used to recruit parent/carers of children diagnosed with autism and aged under 18 attending a mainstream school and who had obtained, were in the process of obtaining or had attempted to obtain an EHCP. Advertisements were placed with local support groups for parents of autistic children, and social-media; snowballing methods were used including word-of-mouth. Seven mothers responded, all meeting the inclusion criteria. One mother was unable to attend an interview due to time constraints; therefore, 6 biological mothers were interviewed and their demographic data collected (see Table 1).

**Table 1.** Participant/child demographic information.

Partiipant	Gender	Age	Marital status	Occupation	Age of child	Gender of child	Health care professional diagnoses	Age at diagnosis	Status of EHCP
Jess	Female	34	Co-habiting	Assistant psychologist	15	Male	^a^Autism Dyslexia	12.5 10	First request refused, currently has EHCP^b^
Ellie	Female	44	Married	Self-employed	12	Male	^a^Autism Hypermobility	_^c^ _^c^	First request refused, currently has EHCP^b^
Lauren	Female	31	Single	Counter staff/carer	6	Male	^a^Autism Hypermobility	5 6	First request refused, does not have EHCP
Jayne	Female	_^c^	Married	SEN co-ordinator	9	Female	^a^Autism	4	First request refused, currently has EHCP^b^
Emily	Female	39	Married	Education advisor	7	Male	^a^Autism Allergies	6 4	Just submitted assessment request
Annette	Female	39	Married	Self-employed	10	Female	^a^Autism Hypermobility Dyslexia Mental health condition	9 8 9 8/9	First request accepted, awaiting EHCP

*Note:* EHCP: Educational Health and Care Plan.

^a^ Autism spectrum disorder.

^b^ Assessment offered after parents began tribunal process (did not go to tribunal).

^c^ No response when asked.

### Participants

Interviews were conducted *via* zoom lasting 30–65 min (*M*** ***=*** **50, *SD*** ***=*** **11.18). Mothers were aged between 31 and 44 years (*n* = 5; *M*** **=** **37.4, SD = 5.03 years), and lived within 3 regions in the south-east and south-west of the United Kingdom (UK). Their autistic child was aged between 6 and 15 years (*M*** **=** **9.83, SD = 3.31); and received their autism diagnosis aged between 4 and 12 (*n* = 5; *M*** **=** **7.2, SD = 2.93). In terms of accessing an EHCP, 66.6% of parents were refused an EHCP assessment on their first request (one did not pursue the EHC assessment; three attained the assessment after parents requested a tribunal appeal).

### Materials

To ensure appropriateness of questions, the interview schedule was developed through consulting previous literature on similar topics around education and support for autistic children and consultation with parents of autistic children with lived experience of accessing an EHCP. Questions included:

- What were your reasons and hopes for accessing an EHCP?
- What was your experience like in trying to obtain an EHC assessment?
- When you think back to your experiences of trying to obtain or maintain your child’s EHCP, was there anything you found particularly helpful or unhelpful?
- In what ways has the EHCP process supported or impacted your own wellbeing?
- What do you think could be changed/needs to stay the same to support parents in accessing an EHCP for an autistic child?

### Ethical considerations

Approval was given by the institutions Ethics Committee (protocol number: LMS/PGT/UH/04995). Confidentiality was ensured and participants gave written informed consent after being told the purposes of the study, their right to withdraw, the recording of interviews and anonymization of quotes used in a publication. Identifiable details were removed, and pseudonyms applied during transcription with recordings deleted thereafter. Following the interviews, participants were thanked and given a debrief sheet which included contact details for further support.

### Analysis

Following transcription, the second author read and re-read the transcripts to become familiar with the data and coded the transcripts; initial themes were derived from the codes. Reflexive conversations with the first author were conducted throughout the interviews and analysis to refine the themes and a theme table constructed with supporting quotes (Braun and Clarke 2006, 2019). Participants were sent the theme table; one adaptation was requested by one mother to add emphasis around legal/illegal aspects within the EHCP process (Treharne and Riggs 2015). All authors, which included those with lived experiences, agreed the final thematic structure and quotes utilized in the paper (O’Brien *et al.* 2014).

## Results

Following the TA analysis, four main themes were developed with ten subthemes. These encompassed mothers’ experience including helpful or unhelpful impacts of the EHCP process (see Table 2).

**Table 2.** Main themes and subthemes.

Main Themes	Subthemes
Barriers for accessing educational support	Accumulation of school-based stressors and managing judgments
	Eruptions at home: The impact from masking at school
The process of obtaining an EHCP	Autism diagnosis: the golden ticket for an EHCP
	The overwhelmingly difficult EHCP process
	Broken trust: wider systems excluding and working against mothers
	Finding supportive allies and building knowledge
Impact on mothers	Maternal stress from the EHCP process
	Worry for their child’s future
Trying to find glimmers of hope	The value of teacher support
	Hope: don’t give up

*Note:* EHCP: Educational Health and Care Plan.

### Barriers for accessing educational support

All mothers’ believed the accumulation of stressors at school meant schools struggled to support their child’s autism, resulting in heightened distress within their child, compounded by their child masking their autism there. Mothers believed teachers tended to blame or judge them for their child’s needs and behaviours necessitating the need to access additional support *via* an EHCP.

#### Accumulation of school-related stressors and managing judgments

Some mothers noted a deep impact from their children facing stressors at school; prior to school being stressful for her child, Ellie stated: ‘I had a lovely, amazing, energetic, happy, intelligent, academic, boisterous, funny, boy, and then I didn’t…then I didn’t, and it got worse and worse and worse and worse and worse’ (Ellie). The repetition of ‘worse’ five times emphasized a cumulative never-ending impact from the stress, Ellie concluded: ‘he went from my boy to my boy with a lot of damage. It happened very quickly’. One can only wonder if the damaging impact from this accumulation of stress could ever be undone, and what impact this might then have on Ellie’s own wellbeing as she witnessed her child’s deterioration yet could not find ways to support him at school. Indeed, Jess noted: ‘it hurts me watching him having to go through it on a daily basis’ (Jess). Some mothers were clear that the stressors were traumatic:

I just want a child who is not doomed from the start…the suicide rates in neurodivergent people is really, really high and that boy was headed in that direction…I just wanted him to have a normal life… a life that wasn’t traumatizing him every day. (Ellie)

The position and support offered by teachers resulted in assumptions made that their child’s behaviour was due to their parenting: ‘they look on it “well, if the parents have done training courses”, jeez why is the emphasis always on us?’ (Emily). Teacher judgments triggered a complex interaction around blame culminating in self-blame: ‘I have been judged…it was…put on me…my parenting…I was blaming myself, like they were blaming me’ (Jess). Accessing a legal framework to support adaptations for their child’s autism and educational needs seemed a way of redirecting judgement, blame and dismissiveness of their maternal opinion: ‘it’s all “mum says”, I think the EHCP is a way of avoiding some of that, “mum says”’ (Annette).

#### Eruptions at home: the impact from masking at school

Some mothers indicated that schools should understand autistic children might present differently at home and school, struggling to communicate needs at school which impacted their ability to manage expected demands in the school setting: ‘they don’t understand that children mask throughout the day…or hold it in…then…come home, they literally erupt’ (Jess); ‘I know they don’t see his violent outbursts…he holds it in…he went on a school trip…he was absolutely hell when he come home’ (Lauren); ‘She comes with the typical challenges of being an autistic girl…a lot of her struggles are internalized and school often wouldn’t know about it’ (Jayne). Indeed, with their child internalizing struggles when at school, the home seemed a safer place to eventually release the pressure build-up and communicate through emotional outbursts. Some mothers named this process at school as masking, a psychological process used to suppress impacts from managing demands or expectations. Consequently, one mother felt if they stated their child was struggling, schools should make adjustments to school-based demands to better match their child’s needs:

…having autistic children who…can mask and do mask…refuse to believe there’s a problem and put anything in place until it goes horribly wrong…so having an EHCP with it in black and white…rather than…trying to convince the SENCO at mainstream secondary that…she sound’s okay, she’ll talk to you like she’s okay, but actually, she’s threatening to kill herself. (Annette)

### The process of obtaining an EHCP

All mothers faced many barriers during the EHCP process, which ultimately necessitated gaining knowledge of SEN and EHCP processes from external sources.

#### Autism diagnosis: the golden ticket for an EHCP

Professional and teacher misunderstanding about their child’s needs meant autism was diagnosed late for some of the children: ‘he wasn’t diagnosed till quite late…the school didn’t think that it was autism…the doctors didn’t think it was autism…he really did struggle with school’ (Jess). There seemed a collective professional dismissiveness of maternal concerns around the impact of undiagnosed autism for her child. Compounding this was the view that proof was essential in attempting to access an EHCP: ‘unless they can actually see your disability or your physical need…they’re like, you need to prove it’ (Lauren). Indeed, accessing an autism diagnosis: ‘was like a golden ticket…I did try before but…they just refused to assess him’ (Lauren). Emily believed without an autism diagnosis her child would not have been considered for an EHCP:

We were discussing this just after he was formally diagnosed, that was…a trigger to them. They felt that…could…secure the deal…you’re being told continuously you need this magic card…someone saying yeah…what you all think is correct…now that’s happened, we can do all of this stuff, stroke, nothing!…it was all hinging on the diagnosis.

#### The overwhelmingly difficult EHCP process

Accessing support *via* an EHCP, was seen as a time-consuming lengthy process:

…they [LA] conceded in January, they agreed she needs an EHCP, because the plan is not finalised, they haven’t been able to do any banding…So they have not been able to recruit any extra resource to support [child] in September. (Annette)

Perhaps this inaccessibility was due to a lack of resources in education, implicated when Annette stated the LA ‘conceded’. Emily was more explicit about this:

I'm really going to have to…illustrate this…protected characteristic…having an EHCP was that, I also hoped we might be able to access…this imaginary provision that exists…where kids who are capable of working at a mainstream curriculum…are supported, but that provision doesn’t really exist educationally. (Emily)

Language such as ‘imaginary’, ‘does not really exist’ suggested it was almost a mythical fantasy, like an intangible crock of gold at the end of a rainbow, which still required attempts to access for their child’s wellbeing. Yet it seemed harder than it needed to be, with an ongoing, battle-like experience to access support implicated: ‘my children…need additional care and attention and take up more time, the thing that is most soul destroying is…fighting with statutory services’ (Annette).

With their child still struggling at school and life being a challenge, diplomacy was essential; it seemed mothers feared irritating staff if due care and attention was not applied when negotiating individualized adjustments for their child:

I felt like I was apologizing for asking…maybe what I'm asking is unreasonable and of course it isn’t…but…I feel you spend a lot of your time like trying to be like on eggshells, you’re being diplomatic, […] not be an inconvenience or giving cause to be annoyed with you or make your life any more challenging. (Emily)

The intensifying battle to access an EHCP resulted in parents feeling apologetic for even asking. Yet mothers were often reaching crisis point, and in this scenario, they found the EHCP process overwhelming and ‘quite harrowing’ (Ellie). Indeed, with so many hoops to jump through, it seemed designed to put parents off pursuing a legal framework for support: ‘…you’re doing EHCP because you’re in absolute crisis…looking at the form it’s so many boxes, it could put a parent off.’ (Emily). Jayne echoed this: ‘It’s time consuming…it’s irritating…I suspect puts off a lot of parents it’s quite intimidating…it’s so complicated…like they’ve taken the process and decided to make it as complicated as possible’. Furthermore, Jess stated: ‘I've had very negative experience with it all…still going through that…negative experience with the EHCP’. Jayne noted: ‘these are…families that are already very vulnerable…with very vulnerable children, some of the most vulnerable children in society…they’re already on their knees…then being asked to access a very cumbersome and intimidating process’. Given the thrice repetition of ‘vulnerable’ Jayne clearly indicated her concerns for children and anyone undertaking the EHCP process – perhaps she implied her own exhaustion when she said parents were ‘already on their knees’.

In trying to access an assessment for an EHCP some parents experienced a court hearing, implicating an on-going battle-like process:

…we still had to have a hearing…we’re looking down the barrel of another ’cos [child’s] EHCP isn’t…finalised, it’s now way overdue but it isn’t actually a finalised plan…we know that with secondary transfer we’re 90% likely to have to go back and fight again … I don’t know whether it’s better or worse when you know what you’re getting yourself into. (Annette)

#### Broken trust: wider systems excluding and working against mothers

All parents acknowledged the legal framework around the EHCP and systems implementing support seemed to work against them:

…primary schools don’t tell you anything…they are legally allowed to do so much…they go, ‘oh no, that’s not our job’…you get passed from pillar to post an awful lot…’I'm going to take you to court’ and they’re like ‘oh fine…I'll see you tomorrow’. (Ellie)

Schools were sometimes reluctant to provide support: ‘…there’s been a lot of resistance by school to get services involved…There’s been a reluctance to get any EHCP, there’s been a reluctance to do any kind of, when I've pinned them down’ (Emily). The repetition of ‘reluctance’ and terms such as ‘resistance’ and ‘pinned…down’ evocatively emphasized how hard it was yet also the importance of pressurizing and fighting within the process.

With an EHCP sitting within a legal framework some mothers, such as Jayne, emphatically highlighted her belief that the LA might be acting unlawfully: ‘Local authority’s the problem!…some of their behaviour, it’s…utterly inappropriate…they go from the outright unlawful…refusing EHCP’s for reasons they shouldn’t, producing EHCP’s that aren’t fit for purpose, to actually…they’ve lied’. Jayne further stated that: ‘Someone somewhere is making that decision not to follow it…trying to make them do what’s lawful, is…very, very complicated…and it’s intimidating’. Annette also felt there was intent behind the process being ‘so strategic, but it comes back to…the other side won’t play by the rules’. Annette clearly drew out the contrasts within the interpretation of the legal framework and how EHCPs were currently being implemented:

My interpretation of the law, SOS!SEN’s [SOS! Special Educational Needs] interpretation of the law, IPSEA’s [Independent Provider of Special Education Advice] interpretation of the law is rather different to [region] County Council staff interpretation of the law…that is really hard because you are dependent on this legal framework, which is meant to ensure that your children will get what they need. And they [LA] just ignore it all the time.

Such experiences around supporting vulnerable children led to broken trust in the system: ‘it’s the only public service where you genuinely cannot trust what you’re being told’ (Jayne).

Parents felt excluded from the process: ‘I’m out of the loop with them’ (Jess); ‘I signed the form…the final submission I didn’t see at all’ (Emily). Ellie described knowing they were in the system, without knowing what was happening: ‘they just go, “we’re dealing with you, you’re in the system” they’re just dragging you along by your feet on a horse…you’re just going with them, but you don’t know what’s happening.’ (Ellie). The term ‘dragging’ evoked a sense of utter powerlessness for mothers, with professionals controlling the system and mothers within it.

#### Finding supportive allies and building knowledge

Despite mothers knowing they were resourceful and capable, knowledge was sought from external organizations to teach and support them through the complex EHCP process: ‘I’m, quite good at doing stuff, it got to the point where I was like…I can’t do this. I can’t do this. So I've paid someone to do it…It’s impossible to understand if you don’t have someone guiding you through it. It’s impossible!’ (Ellie). Ellie’s repetition of ‘It’s impossible’ emphasized the extremity of how difficult supporting her autistic child through mainstream education was. Indeed, Annette compared the process to an exam with no guidance:

When you go through it with someone who knows what they’re talking about, these…questions are…looking for things that aren’t what the question says…It’s like an exam, you need to know what you’re meant to put in each box…It’s just impenetrable if you don’t have someone who’s been there before…I find that…frustrating.

Again, the language highlighted the extremity of difficulties within the system (‘impenetrable’, ‘frustrating’). Consequently, as the battle to access an EHCP intensified, mothers sourced SEN allies supporting parents engulfed in the EHCP process:

…feels…very disconnect…I'm sure SENDIASS [Special Education Needs and Disabilities Information, Advice and Support Services] are hugely busy…it just feels really contradictory…these are [region] kids in [region] schools, [region] SEN system and you have to go somewhere else to try and fight you, fight the system that should be delivering (Emily)

There was an almost incredulous tone, that mothers had an exhausting fight with the local system tasked with supporting children with SEN. Mothers joined groups to gain answers: ‘[I] belong to all the different groups and…they’ll make a question and they’ll put it in all the groups and somebody will know the answer. That’s how we get by’ (Ellie). For some it triggered self-directed learning: ‘I enrolled on a psychology course…to be able to…understand his disability a little bit better…I’m 7 years down’ (Jess). In a supportive giving-back process, some educated themselves on support processes, SEN and EHCPs to then support other parents:

There are parents like myself…who are starting to train and offer help to people, […] it absolutely feels at this stage where it’s becoming the responsibility of families of children with SEN to take care of other families…It’s becoming a pay it forward thing rather than actually there being a safety net for them within society. (Jayne)

Clearly mothers were dedicated to supporting their children through mainstream education despite the process being ‘soul destroying’ (Annette). In such desperate circumstances, one mother took extreme measures, perhaps symbolizing her despair yet also the extent of her concern and care to do right by him: ‘I called Social Services on myself…the doctors wasn’t helping me the school weren’t helping me…I needed some kind of help to manage his behaviours’ (Jess). Perhaps if earlier support had been forthcoming this action might have been unnecessary.

### Impact on mothers

This theme outlines the impact from accessing support for their children, exacerbating worry for their child’s future and maternal stress.

#### Maternal stress from the EHCP process

Mothers repeatedly reported how stressful accessing an EHCP for their child was: ‘it was stressful…getting told no’ (Lauren); ‘school were causing me so much stress’ (Jess); this had a detrimental impact on mental health: ‘It’s really stressful…I don’t get stressed…This is the…first time in my life that I've gone right…I'm gonna pay someone now, I have to because I'm capable…This is impossible…Awful mental health’ (Ellie). Emily echoed this:

It hasn’t really supported my own well-being…it’s added to my anxiety…made me feel deskilled on occasions…it’s caused me a lot of anger…with the school…I felt quite frustrated about it…it sounds a bit severe depression, but you can kind of come away feeling this is insurmountable, we’re never gonna get over this…whole thing. And then even if we do get the EHCP, they probably won’t do anything with it.

The fight for support drained all available resources implicating a sense of unshakeable battle fatigue and ‘insurmountable’ defeat. Indeed, Annette noted: ‘The process has not supported my own wellbeing…just a huge detriment, that sucks our…energy’. As the journey progressed the culmination of each step accumulated and multiplied the stressors: ‘you’ve got the stress of trying to get a diagnosis followed by the stress of an EHCP’, thus, the process ‘could quite easily tip people over the edge’ (Jayne). This impacted everyday activities, leaving mothers dreading life:

I was a wreck […] I dreaded him coming home from school. I dreaded waking him up in the morning because…I wasn’t getting support. I wasn’t being supported. He wasn’t being supported…it was like a big cloud constantly over me. (Jess)

Not only did mothers have to manage the trauma-like experiences their children were having, but their own lives also had to shift with the time the EHCP process demanded:

…my whole world especially the people that I can talk to, had to change…no matter how good your other friends are, if they don’t know what an EHCP is, and you’re utterly consumed by this stuff…it’s not the same as other people who you might have less in common with in other ways. (Annette)

There was a sense of isolation and loneliness which seemed to fill the space which the EHCP occupied, and which had previously been filled with friends.

#### Worry for their child’s future

With mothers worrying about their child struggling at school, this also extended to worrying about their child’s future: ‘it’s the catastrophes about the future…has impacted on us’ (Emily). This worry extended further beyond education:

…how is he going to be affected by…not sitting in exam not having…additional support, not being able to learn what he should be…if he wants to go to college… he’s not going to be aware of what is expected. (Jess)

The wealth of the worries of how their children might navigate life and its demands, beyond the present and going into the future, seemed to generate a heavy burden for mothers. These worries were also linked to past experiences, for example, Emily was concerned with the upcoming transition to secondary school as her son had previously shown regression when life demands were too great: ‘we then get in adolescence, a much tougher time of life…with my own son, I've seen some regression between three and seven…that’s something we’re going to have to navigate’. The transition to secondary school was also a concern for Annette:

I don’t think that matters so much at primary…but it’s probably going to matter more in a couple of years…the anxiety, the build-up that stops her enjoying and…participating in things other people might without thinking about it. (Annette)

Compared to others, Annette could already see more anxiety her child might have to experience. Perhaps worries intensified when the EHCP did not meet expectations: ‘There’s still…what next after the EHCP because…I'm not sure it actually improves outcomes for autistic kids, really? Not sure what it does in real terms’ (Emily). Some mothers felt that schools similarly experienced this: ‘…they were just struggling to implement, because it wasn’t clear…it was like, this is useless. Like what are they supposed to do with this? We’ve got these really vague instructions…we need…more clarification around this’ (Jayne).

### Trying to find glimmers of hope

Despite the stressful nature of the EHCP process, some mothers identified glimmers of hope.

#### The value of teacher support

Beyond the stressful EHCP some mothers found oases within invaluable pockets of individual care, dedication and support from some teachers: ‘although he isn’t getting the support he’s actually entitled to, the two teachers…that do have kind of his best interests at heart are what make his school beneficial for him’ (Jess); ‘She’s excellent, she knows her stuff…she’s excellent!…she’s really trying to help’ (Ellie); ‘although it’s not always within school’s control, the difference when you’ve got a good, experienced teacher who gets it, it’s so huge for both her and us’ (Annette).

Mothers acknowledged sometimes support for autistic children was out of school’s control, but expressed teachers support was valued: ‘they’ve always worked with us, but…her needs were definitely above and beyond what they could manage on their own’ (Jayne). These additional needs were why mothers ultimately had to commence the EHCP process.

#### Hope: don’t give up

Parents believed their child would gain more if more was provided, however, changes in funding streams meant funding was still unavailable once the EHCP was in place: ‘her EHCP doesn’t have any funding…they’ve done what they can but without any extra resource, they are…a bit stuck.’ (Annette). Similarly, one school was emphatic they could not meet a child’s needs without additional funding: ‘the head teacher has said no, we’re not meeting [child’s] needs without additional funding, we can’t’ (Annette). Thus, mothers seemed to face a no-win situation, exacerbating their worries and the burdens they were carrying.

Nevertheless, half of mothers felt hopeful that an EHCP might enable their child to better manage mainstream education: ‘I thought he would benefit from an EHCP’ (Emily); thus, Emily pushed, asking school ‘“what needs to happen for the EHCP…’cos I'm really desperate and I feel like it would be supportive”’. Jess stated she ‘hoped it would give him access to the support he’s actually entitled to…a chance to catch up on work that he’s missed…I just hoped it would give him that opportunity that to do the best he can in an education setting’. Furthermore, Emily stated: ‘I was hoping it was going to…safeguard his future and preventing exclusion…I also hoped it’s going to give some funding for a one-to-one’. Indeed, the EHCP provided some protection for Jess’s child being excluded: ‘I think the EHCP…hasn’t really done anything, if anything it’s the only thing keeping him from being kind of kicked out of school permanently’ (Jess).

This hope for an inclusive education, meant mothers stuck with a difficult process, indicative of resilience and determination even in a barren landscape seemingly void of real opportunities; for example, Jess reflected that educators’: ‘should make sure that my child…any child…with a diagnosis or an EHCP has the same opportunities as any child’; adding that: ‘…from getting that diagnosis…his EHCP was put into place…I felt a little bit more confident in him being in that environment, knowing that he was going to get support where he needed it’, yet she concluded with a tone of sadness: ‘but it wasn’t like that’.

In contrast just one mother reported a more positive outcome: ‘Life is a lot easier since we’ve got the plan…partly because she now has consistency and routine so she struggles less’ (Jayne). The hopefulness that they might have a positive outcome meant three mothers retained a resilient optimism, giving words of encouragement even whilst facing adversity: ‘just don’t give up…keep going back, they’re soon going to get sick of it’ (Lauren); ‘don’t give up…don’t try and do it on your own because they will muck you around’ (Jayne); ‘Don’t give up. Do not give up, you know your own child. It might take a year it might take two years…but keep at them, keep chasing them’ (Jess).

## Discussion

The SEN code of practice includes the requirement of early identification as one of its key principles (DfE 2015); with diagnosis being important for the child’s mental well-being through helping children to understand the reasons for their difficulties (Howes *et al.* 2021). However, in line with previous research (Tissot 2011, Galpin *et al.* 2018), all mothers reported their autistic child struggled to manage the diverse demands encountered within mainstream education settings without appropriate educational support and adjustments. Consequently, mothers battled the education system for necessary support (Galpin *et al.* 2018); for mothers whose children were about to transition to secondary school, this intensified the battle given their concerns around the impact of this transition, a known difficulty for autistic children and their families (Tso and Strnadová 2017).

Some mothers stated their child was traumatized from trying to *manage the accumulation of school-based stressors*; and believed their child’s difficulties were beyond the expertise of some of their teachers’ abilities. Consequently, issues were not addressed, and too often *judgements* and/or blame for their child’s behaviour and emotional state were directed toward mothers (Jackson *et al.* 2020). Such judgments were unhelpful, especially as children’s attainment can be affected when education providers are less positive about SEN (Ellins and Porter 2005). Despite autistic children being at greater risk from anxiety and depression (Lai *et al.* 2019), the inability to access educational support was compounded by some children *masking at school* so that teachers were unable to see their additional needs. Instead, the children *erupted at home*, displaying heightened distress in the form of meltdowns. The impact of masking autism has been further implicated in lifetime suicidality in autistic students (Cassidy *et al.* 2020).

Consequently, with their child not developing like their peers, mothers’ fears about the present extended into *worries for their child’s future*, intensifying the need for better educational support. However, a key barrier to accessing support was lack of provision; indeed, 82% of mainstream schools state they have inadequate funding to support SEN students (The Key 2016). With educational needs of autistic children not always being met (Russell and McCloskey 2016), mothers negotiated and battled to access more extensive educational support *via* an EHCP (GOV.UK 2023).

In this study, the context of limited provision meant accessing an EHCP was viewed as a magic card requiring persistence and resilience to access. It also represented *somewhere over the rainbow*, a place where additional provisions could be acquired in the hope their child could thrive again. However, additional systemic barriers were evident, for example, with *autism diagnosis* viewed as *the golden ticket,* some mothers had to jump through diagnostic hurdles before starting an EHCP application. With extensive waiting lists and difficulties accessing diagnostic assessments (Hayes *et al.* 2022), this can substantially delay children accessing the adjustments they need, making the whole process more time-consuming and cumbersome.

Compounding these delays were LA gatekeepers presenting multiple roadblocks through controlling the *difficult EHCP process*. Mothers were left with *broken trust* with *wider systems excluding and working against them*; ultimately evoking a sense of powerlessness. Of note, there was a higher proportion of mothers initially being refused an EHC assessment compared to previous surveys (66% compared with 42%; NAS 2017). A high proportion of mothers then requested a tribunal, and one mother went to court to battle with the LA. This seems to reflect reality with a 20% increase in tribunal outcomes between 2021 and 2022; of note 96% of outcomes eventually made by the tribunal favored the appellant (DfE 2023), likely the parent/carer.

Following the EHC assessment, for some mothers the LAs resistance continued by delaying the provision of necessary adaptations, not giving anything further, or providing vague adjustments, which the school struggled to implement. Some mothers felt LAs deliberately made the process difficult to discourage parents from applying, with some tactics felt to be unlawful. *Maternal stress from the EHCP process* was exacerbated by its time-consuming, intimidating and complex nature which resembled a battleground taking all their resources and energy. Worries about their child intensified, detrimentally impacting their own mental health, such that some began to dread life. With no crock of gold at the end of the rainbow, for most mothers their initial hope turned to despair, with a sense left that accessing provision through an EHCP was a mythical quest, mirroring recent research highlighting the impact obtaining an EHCP can have on families (Starkie 2023).

The accumulation of stressors their child experienced at school continued and seemed to mirror the mothers’ accumulation of stressors to access educational support, often an insurmountable journey described as ‘harrowing’ and ‘soul destroying’. There was no real space left to connect with friends, adding to the sense of isolation and loneliness mothers experienced (Galpin *et al.* 2018). Perhaps it is unsurprising that mothers of autistic children are at greater risk from depression (Singer and Floyd 2006); and, compared to mothers with typically developing children, intellectual disability, and specific learning disorder, mothers of autistic children report the highest levels of stress (Alamdarloo and Majidi 2022).

Despite the presence of a metaphorical battleground, some mothers *valued teacher support* from some teachers (Hasson *et al.* 2022). Furthermore, an EHCP provided some with a buffer to the judgments and dismissiveness some had experienced from teachers by verifying their child’s autism and additional needs. For one mother, the EHCP provided the stability and consistency their child needed in education. Two mothers believed the EHCP protected their child from school exclusion; data does suggest children with SEN and EHCPs are less likely to be excluded than children with SEN and no support (GOV.UK 2022). This buffering role is crucial given the impact exclusion from school has on the wellbeing of children and mothers (Martin-Denham 2022).

Mothers also *found supportive allies* from local groups and other parents, *building their own knowledge*. Thus, with love, dedication, and resilient optimism, some retained *hope* that their child might flourish again, providing words of encouragement for others to not *give up*, giving back their knowledge to others encountering similar battles (Cleary *et al.* 2023). However, one is left with the sense of what happens when mothers do not have the knowledge, ability or resources to endlessly battle with these systems.

It is important to note that our sample may reflect mothers who were struggling with the EHCP process and, therefore, may not account for parents with more positive experiences. Additionally, fathers’ voices were absent from the current research, despite the study being openly advertised to all parents and carers. Father’s views have gathered far less attention in the literature to date, and there is a clear need to understand their thoughts regarding their child’s education (Hartley and Schultz 2015). Furthermore, as the current sample was largely from a south-eastern region in the UK, with one living in the south-west; there may be regional differences, with parents in other areas of the country possibly having better access to EHCP’s. The mothers were also from a highly heterogenous demographic group, with all identifying as white British and of higher social economic status. Nevertheless, qualitative research has been recommended in autism to enrich understanding around presentations and its impact across settings (Adams *et al.* 2019). Future research would benefit from interviewing parents across the country to understand regional experiences, alongside exploring diverse ethnic, cultural and socio-economic backgrounds. Finally, there was evidence of complex physical needs, with half of the children from our sample diagnosed with hypermobility, often identified in autistic children struggling with school demands (Keville *et al.* 2021). This warrants further exploration given recent research is identifying greater pain and dysautonomia from hypermobility in autistic samples and other neurodivergent conditions compared to the general population (Csecs *et al.* 2021).

## Conclusion

Despite schools’ responsibility to ensure all pupils are accommodated in mainstream settings (Wedell 2005), clearly the drive for inclusive education has yet to work effectively for many autistic children (Dillenburger et al., 2015). To this end, given many families with vulnerable children are not accessing the support their child needs to manage the demands presented within mainstream settings, rather than LAs being tasked with SEN provision (gatekeeping funding and employing lawyers to manage tribunals), it is critical more productive and effective use of funding streams are set up. A streamlined national and regional service could more effectively manage, educate and allocate provision for SEN within mainstream settings. Stakeholders should include parents, SEN teachers, medical specialists with expertise in complex medical conditions associated with autism, local support groups, policy makers, and supportive organizations specializing in SEN law (such as SOS!SEN, IPSEA and SENDIASS). Ultimately, we need the humanity and collective action to manage autism more effectively in mainstream settings, to enable children to live and flourish with their condition and vicariously support parental wellbeing.

## Data Availability

The data that support the findings of this study are available within the article and on request from the corresponding author. The data are not publicly available due to privacy or ethical restrictions.
